# The Influence of COVID-19 on Influenza and Respiratory Syncytial Virus Activities

**DOI:** 10.3390/idr14010017

**Published:** 2022-02-14

**Authors:** Pritish Mondal, Ankita Sinharoy, Suparna Gope

**Affiliations:** 1 Department of Pediatrics, Penn State College of Medicine, Hershey, PA 17033, USA; 2 Heart and Vascular Institute, Penn State College of Medicine, Hershey, PA 17033, USA; asinharoy@pennstatehealth.psu.edu; 3 Department of Administration and Leadership, Indiana University of Pennsylvania, Indiana, PA 15705, USA; hdhv@iup.edu

**Keywords:** influenza, RSV, COVID-19, flu vaccine, pandemic

## Abstract

**Background:** Respiratory viral diseases have considerably declined since the COVID-19 outbreak, perhaps through influence by nonpharmaceutical interventions. We conducted a cross-sectional study using the CDC database to compare the pre- vs. post-pandemic flu activity (incidence) between the US states. Our secondary objectives were to estimate the association between flu activity and flu vaccination rates and compare the national trends of flu and RSV activities since the pandemic outbreak. **Methods:** We estimated the difference between pre-pandemic (April 2019–March 2020) and post-pandemic (April 2020–March 2021) flu activity between individual states using the Wilcoxon signed-rank test. The association between flu activity and immunization rates was also measured. Finally, parallel time trend graphs for flu and RSV activities were illustrated with a time series modeler. **Results:** The median (IQR) pre-pandemic flu activity was 4.10 (1.38), higher than the post-pandemic activity (1.38 (0.71)) (*p*-value < 0.001). There was no difference between pre-pandemic (45.50% (39.10%)) and post-pandemic (45.0% (19.84%)) flu vaccine acceptance (*p*-value > 0.05). Flu activity and vaccination rates were not associated (*p*-value > 0.05). Flu activity has declined since the COVID-19 outbreak, while RSV made a strong comeback in June 2021. **Conclusion:** Flu activity has significantly diminished throughout the pandemic while a sudden upsurge in RSV is a public health concern indicative of possible resurgence of other viruses. Flu vaccine acceptance neither changed during the pandemic nor influenced the diminished Flu activity.

## 1. Introduction

In March 2020, COVID-19 was declared a pandemic by the World Health Organization [1]. Since then, most countries have implemented preventive guidelines, including quarantine, social distancing, hand washing, and face mask usage in public places [2,3]. Although these measures were primarily implemented to prevent the spread of COVID-19, incidences of other respiratory viruses were also influenced by these nonpharmaceutical interventions [4,5]. Influenza (flu) and RSV are common human respiratory viruses, which traditionally cause significant mortality and morbidity [6]. An average of 28 million people were affected and 35,000 people died of flu annually in the USA in 2010–2020 [7]. In contrast, RSV has been the predominant cause of bronchiolitis and pneumonia in infants and also affects the elderly population aged 65 years and over [8]. While a flu vaccine is available, only a monoclonal antibody can be offered to prevent RSV [9]. Flu immunization reduces the risk of having flu by 40–60% [10]. A national survey from 2020–2021 suggested that the people vaccinated against flu were more likely to accept the COVID-19 vaccine, too [11]. However, to the best of our knowledge, the flu vaccine acceptance trend at the time of the pandemic has not been well-reported in the USA.

The Centers for Disease Control and Prevention (CDC) publish weekly flu activity reports for every state. However, it is difficult to extract and comprehend the difference in flu activity between individual states from that vast database. Thus, a US political map with categorical highlights of individual states based on recent flu activity would perhaps be helpful to visualize the variation across the country. This variation in flu activity might be influenced by factors such as nonpharmaceutical interventions [12,13], while flu vaccine acceptance rates could also be another contributor [14].

RSV and flu cases declined drastically in 2020 [15,16]. However, RSV made a strong comeback in 2021 [17]. Although data on flu and RSV activities are available in the public domain, a comparative analysis between the trends and timelines of flu and RSV since the COVID-19 outbreak has not been well-described in the literature. From the public health perspective, understanding the variation in their trends would be of interest since the two viruses differ in the incubation period, mode of transmission, and period of infectivity [18].

We conducted a cross-sectional study based on the data publicly available on the CDC website to compare the pre- vs. post-pandemic variation in flu activity between the fifty US states and present the findings on a US political map that could be readily visualized. The secondary objective was to estimate the association between the flu activity and the flu vaccine acceptance rate since the pandemic outbreak. Finally, we compared the national trends in flu and RSV activities over the last two years (2019–2021) and presented them as a parallel time trend graph.

## 2. Materials and Methods

### 2.1. Data Collection

We accessed the CDC database and collected weekly flu activity reports from individual states [19]. Information regarding flu vaccine acceptance was collected from the FluVaxView interactive website [20]. We further accessed the National Respiratory and Enteric Virus Surveillance System (NREVSS) [21] in October 2021 and collected data on RSV incidence. RSV activity reports from the last two years (from November 2019 to October 2021) were publicly available on the NREVSS website at the time of data collection.

### 2.2. Data Analysis

(a) Flu activity was reflected on the CDC website on a severity-based scale with 13 levels. In comparison, RSV activity was reported as the number of new cases. We normalized RSV cases on a “1–13 scale” to match with the flu activity and run comparative analysis. The flu vaccine acceptance rate represented the percentage of the immunized population (individuals aged six months or above). The flu immunization season lasts from July of the given year to May of the following year. The flu vaccine acceptance rate (percentage of the population) increased cumulatively in every passing month.
(b) The difference between pre- (from April 2019 to March 2020) and post-pandemic (from April 2020 to March 2021) flu activity between individual states were compared using Wilcoxon signed-rank test (nonparametric data). Fifty US states were divided into five quartiles (cohorts) depending on the decline in flu activity. An online graphics tool was used to build a US political map [22] demonstrating five cohorts of US states based on the decline in flu activity.
(c) Spearman’s correlation analysis (nonparametric data) estimated the association between monthly flu activity and flu vaccination rates.
(d) We compared both RSV and flu activities between the peak season in 2021 with the corresponding weeks of 2020.
(e) Time trend analysis: since the incidence of respiratory viral infections has changed considerably since the COVID-19 outbreak, a time trend analysis was conducted for flu and RSV activities (between November 2019 and October 2021). We used the SPSS time series modeler [23] to generate a time trend graph for the parallel representation of both viral diseases.

## 3. Results

**Figure 1:**
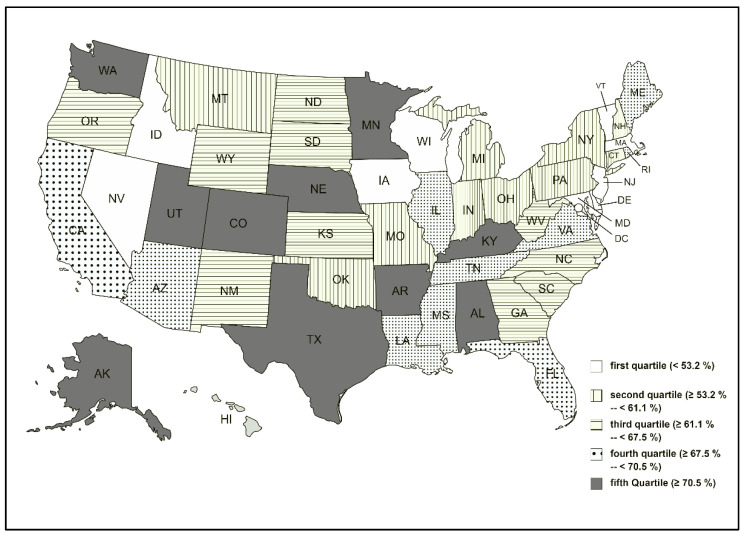
Average activity changes between the pre-COVID-19 (from April 2019 to March 2020) and post-COVID-19 (from April 2020 to March 2021) months.

**Figure 2:**
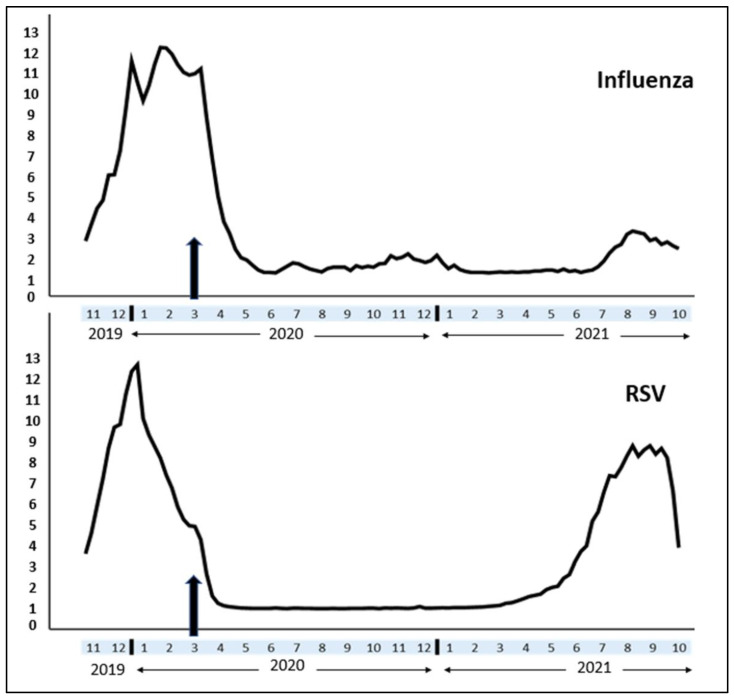
Seasonal variation in flu and RSV activity (1–13 scale) over the last two years (from November 2019 to October 2021). The vertical arrow indicates the time when COVID-19 was declared a pandemic.

(a) The median (IQR) value of monthly flu activity during the pre-COVID months (from April 2019 to March 2020) was 4.10 (1.38) compared to the post-COVID months’ (from April 2020 to March 2021) activity of 1.38 (0.71). The difference was statistically significant (Wilcoxon test: *p*-value < 0.001). Table 1 demonstrates pre- vs. post-COVID-19 flu activity in all US states, while Figure 1 categorizes the US states based on the rate of decline in flu activity. The variations between the states were random, and we did not find a trend according to the US regions.
(b) Pre-COVID-19 flu vaccination rate (% of the population) 45.50% (39.10%) was identical to the post-COVID-19 vaccine acceptance rate 45.00% (19.84%), and the difference was not statistically significant (Wilcoxon test: *p*-value > 0.05).
(c) Flu activity and vaccination rates did not have an association either during the pre-COVID-19 (Spearman’s correlation coefficient = −0.141, *p* = 0.329) or post-COVID-19 period (Spearman’s correlation coefficient = 0.023, *p* = 0.875).
(d) Peak activity season: RSV activity considerably increased since mid-June 2021 (Figure 2). The mean (SD) weekly RSV activity between June 2021 and October 2021 was 6.24 (2.34) higher compared to the RSV activity (0.02 (0.01)) of the corresponding weeks of 2020. However, the average flu activity between June and October 2021 (1.90 (0.62)) was relatively stable compared to the corresponding weeks in 2020 (1.17 (0.12)).
(e) Time trend analysis: since COVID-19 hit the USA, both flu and RSV cases declined significantly in 2020. While flu incidence continued to remain low till October 2021, an untimely RSV peak occurred earlier than the traditional RSV season and remained elevated till October 2021 (Figure 2).

## 4. Discussion

This cross-sectional study based on weekly flu activity (2019–2021) in all US states re-emphasized the post-pandemic decline in reported flu cases. We also found that flu vaccine acceptance was primarily unchanged between the pre vs. post-pandemic periods, and the vaccine acceptance rate did not influence the flu activity. Finally, the time trend analysis illustrates a low flu profile throughout the pandemic (till October 2021), and peak RSV activity in the summer of 2021.

Several studies conducted during the early months of the pandemic demonstrated an overall decline in non-COVID-19 respiratory viral infections [24,25]. Reports from other countries, including China, Australia, and France, also cited diminished flu cases since the pandemic was declared [26,27]. Stamm et al. described a parallel decline in flu and RSV incidence in Germany in 2020, while Tempia et al. reported a similar trend in South Africa [28,29]. Likewise, both flu and RSV cases declined in the USA in 2020. Flu vaccine acceptance behavior is known to be influenced by COVID-19. A UK-based national survey reported that the pandemic had motivated unvaccinated people to accept the flu vaccine [30], while another survey from Pennsylvania, USA, described net favorable changes in flu vaccine acceptance behaviors since the COVID-19 outbreak [14]. However, when the data from the CDC were analyzed, we found an unchanged flu vaccine acceptance rate since the pandemic outbreak. A sudden uptrend in RSV during COVID-19 was previously reported, mostly from regional hospitals [17,31]. In contrast, our study comprehensively summarized statistics from a national database.

Several factors influence the transmission of viral respiratory diseases. Susceptible host, virus, and the environment have a complex interplay which determines the successful spread of the disease [32]. While flu can be transmitted by contact, droplet, or aerosol, [33,34,35] RSV is predominantly transmitted via direct or indirect contact and often remains transmissible for a more extended period than flu [33,36,37,38,39]. Understandably, nonpharmaceutical interventions such as hand washing, face masks, and social distancing helped to prevent flu, and we found that flu vaccination coverage certainly was not the reason behind remarkable flu containment. However, an untimely resurgence of RSV in 2021 was unique. In the past, the traditional winter peak of RSV helped to build immunity among the exposed children. During the first post-pandemic season, RSV was effectively prevented by nonpharmaceutical interventions [40]. Subsequently, the children became immunologically naïve against RSV, and infants did not receive passive immunity from their mothers either. Moreover, routine immunization is available against flu, but not against RSV. All these factors perhaps led to an early resurgence in RSV in countries like the USA, Australia, and South Africa [41,42,43].

Our study has several limitations. We compared the flu and RSV activities only, while other human respiratory viruses such as rhino-enterovirus, human metapneumovirus, and parainfluenza were not considered. The impact of nonpharmaceutical interventions on non-COVID-19 respiratory viral diseases has been well-accepted. However, mask policy and lockdown practices have changed several times in every state since the COVID-19 outbreak. Thus, direct cause–effect analyses of the preventive practices and flu or RSV activities were beyond the scope of this study. Furthermore, identifying the predictors of RSV resurgence was not the focus of our research. Nonetheless, this study should be of interest among the clinicians since the decline in flu activity was comprehensively summarized on the US political map. The parallel time trend graph of flu and RSV activities should also help visualize the pattern over the last two years. Finally, very few previous studies focused on the flu vaccination trend and its association with flu activity during the COVID-19 pandemic. Thus, our study results could be helpful to epidemiologists.

Although nonpharmaceutical interventions helped to reduce non-COVID-19 respiratory viral diseases, we should not shift our focus from traditional respiratory viruses to COVID-19 only. The upsurge of RSV perhaps was not an isolated phenomenon and should be considered as an eye-opener as other respiratory viruses can have a similar comeback [25]. Some experts have even warned against the future resurgence of flu [44]. It is time for policymakers, stakeholders, and clinicians to be aware of the potential untimely resurgence of viral diseases and be more flexible accordingly. We found significant variations in the decline in flu activity between different states. Future researchers may focus on the potential factors that led to better containment of flu activity in some states and build informed preventive strategies. Finally, in-depth research on the predictors of RSV resurgence would be of importance, as a better understanding of the disease process from the epidemiological and virological perspectives would help to prevent similar circumstances in the future.

## 5. Conclusions

Flu activity had significantly declined since the pandemic outbreak and remained low until October 2021. RSV incidence also declined in 2020; however, it had an untimely peak in summer 2021. Flu vaccine acceptance neither changed during the pandemic nor was associated with declined flu activity. An untimely upsurge in RSV cases is a public health concern and indicates a possible future resurgence of other respiratory viral diseases.

## Figures and Tables

**Table 1:** Pre-COVID-19 (from April 2019 to March 2020), post-COVID-19 (from April 2020 to March 2021), and percentage decline in flu activity between the US states.

State	Pre-COVID-19 Flu Activity	Post-COVID-19 Flu Activity	Decline in Flu Activity (%)
Alabama	5.36	1.21	77.43
Alaska	3.13	0.84	73.16
Arizona	3.73	1.16	68.90
Arkansas	4.44	1.06	76.13
California	3.79	1.21	68.07
Colorado	5.15	1.27	75.34
Connecticut	4.59	1.80	60.78
Delaware	2.01	1.05	47.76
District of Columbia	3.39	2.02	40.41
Florida	4.12	1.32	67.96
Georgia	5.62	2.04	63.70
Idaho	1.80	2.30	−27.78
Illinois	4.46	1.45	67.50
Indiana	3.22	1.34	58.39
Iowa	3.28	2.39	27.13
Kansas	4.43	1.63	63.21
Kentucky	4.67	1.28	72.59
Louisiana	7.42	2.19	70.49
Maine	3.58	1.07	70.11
Maryland	4.38	2.19	50.00
Massachusetts	4.08	1.91	53.19
Michigan	2.57	1.00	61.09
Minnesota	4.07	1.00	75.43
Mississippi	4.77	1.51	68.34
Missouri	3.30	1.51	54.24
Montana	2.57	1.10	57.20
Nebraska	4.50	1.16	74.22
Nevada	2.47	1.50	39.27
New Hampshire	2.43	1.00	58.85
New Jersey	4.65	2.56	44.95
New Mexico	5.13	1.88	63.35
New York	4.25	1.85	56.47
North Carolina	4.15	1.35	67.47
North Dakota	3.81	1.48	61.15
Ohio	2.42	1.11	54.13
Oklahoma	4.95	2.18	55.96
Oregon	4.05	1.57	61.23
Pennsylvania	4.05	1.80	55.56
Rhode Island	3.60	1.02	71.67
South Carolina	5.75	1.89	67.13
South Dakota	3.02	1.05	65.23
Tennessee	5.07	1.41	72.19
Texas	5.71	1.20	78.98
Utah	4.45	1.12	74.83
Vermont	3.28	1.65	49.70
Virginia	5.46	1.52	72.16
Washington	4.71	1.22	74.10
West Virginia	3.65	1.25	65.75
Wisconsin	4.29	3.11	27.51
Wyoming	3.31	1.15	65.26

## Data Availability

Data will be made available upon request.
